# Epidemiology of invasive meningococcal disease and sequelae in the United Kingdom during the period 2008 to 2017 – a secondary database analysis

**DOI:** 10.1186/s12889-022-12933-3

**Published:** 2022-03-17

**Authors:** Sandra Guedes, Hélène Bricout, Edith Langevin, Sabine Tong, Isabelle Bertrand-Gerentes

**Affiliations:** 1 Sanofi Pasteur, 14 Espace Henry Vallée, 69007 Lyon, France; 2 Sanofi, Chilly-Mazarin, France

**Keywords:** Meningococcal disease, Incidence rates, Retrospective observational study, Sequelae, United Kingdom

## Abstract

**Background:**

Invasive meningococcal disease (IMD) causes high fatality in untreated patients alongside long-term sequelae in 20% survivors. For a comprehensive assessment of epidemiology, an analysis of these sequelae is required. This study aims to investigate the epidemiology of disease between 2008 and 2017 including a description of the sequelae, through the analysis of data collected from the UK Clinical Practice Research Datalink (CPRD) linked with data from the Hospital Episode Statistics (HES), and Office for National Statistics (ONS) mortality registry data.

**Methods:**

This was a 10-year retrospective observational cohort study designed to describe the incidence, case-fatality rate (CFR) and occurrence of sequelae due to meningococcal disease, in the UK between 2007 and 2017 using data from the UK CPRD-HES-ONS. Cases were identified and matched on age, gender, date of diagnosis of IMD and followed-up-time with a control group without IMD. Demographics, clinical characteristics, mortality, and IMD-related sequelae were examined for IMD cases and compared with matched controls for a more comprehensive assessment.

**Results:**

The study analysed 640 IMD patients with majority of the cases diagnosed (76.9%) in a hospital setting. Age-group analysis showed a decrease in the incidence rate of IMD in patients aged <1 year (30.4 – 7.5%) and an increase in those >50 years (10.4 – 27.8%). CFR was slightly higher among females, toddlers, and adults >50 years. No significant change in CFR was observed over study period. Case-control study showed a higher number of IMD sequelae among cases compared to age- and gender-matched controls, especially in those ≥ 50 years.

**Conclusion:**

The study showed that, despite a relatively low incidence rate, IMD is responsible for a high CFR, namely in older age groups and by a high number of IMD sequelae. The study showed that leveraging data from existing databases can be used to complement surveillance data in truly assessing the epidemiology of IMD. Despite the availability of routine vaccination programs, IMD still poses a significant burden in the healthcare system of the UK. Optimization of vaccination programs may be required to reduce the disease burden.

**Supplementary Information:**

The online version contains supplementary material available at 10.1186/s12889-022-12933-3.

## Background

Invasive meningococcal disease (IMD), caused by Gram-negative bacterium *Neisseria meningitidis*, is a potentially fatal disease. Nearly 8% to 15% of patients with IMD die even when the disease is diagnosed early, and adequate treatment is started. If untreated, IMD is fatal in 50% of patients and can cause long-term sequelae including brain damage, hearing loss, or disability in up to 20% of survivors [1]. The clinical presentation of IMD is diverse with meningitis and septicemia being the most common modes of presentation. The severity of manifestations ranges from bacteraemia, associated with mild, non-specific symptoms, to fulminant sepsis with multiorgan failure and death. Localised infections (such as conjunctivitis or septic arthritis) as well as chronic disease may be the sole clinical manifestations but can lead to disseminated fulminant disease [2]. Twelve serogroups of *N. meningitidis* have been identified, with six serogroups – A, B, C, W, X, and Y – being responsible for virtually all invasive disease [2].

The epidemiology of IMD is dynamic, with different geographical distributions and varying incidence of *N. meningitidis* serogroups and the emergence of new strain variants [3]. Around 1.2 million people are estimated to be diagnosed with IMD per year, with nearly 135,000 case fatalities worldwide [4]. Although IMD affects individuals of all ages, the highest incidence occurs in young children, with a second disease peak among adolescents and young adults [5, 6]. The incidence is also high in the elderly population, the age group with the highest case fatality rate (CFR) [7–10].

According to the Global Disability-Adjusted Life Years (DALY) estimation, the burden of all-age meningitis from all causes was 20.4 million DALY (range: 17.8–23.4) in 2017 [11]. In younger ages, meningococcal meningitis and other bacterial meningitis are the predominant causes of new cases and deaths. Meningitis and meningococcal meningitis also causes a high burden in the elderly population, with increasing levels of incidence, mortality, and Years of Life lived with Disability (YLD) rates [12].

The most effective approach to prevent IMD is through vaccination [13]. Although the United Kingdom (UK) became the first country in the world to routinely vaccinate against serogroups B and C, the incidence of meningococcal disease across all age groups is still relevant [14, 15]. Meningococcal serogroup C (Men-C) conjugated vaccine was introduced in the UK in 1999, and the cases of IMD fell dramatically by over 90% in immunized age groups and indirectly, by two-thirds in other age groups due to reduced carriage and exposure. The emergence of serogroup B and serogroup W led to the introduction of meningococcal serogroup B (MenB) vaccine in infant immunization schedule in 2015 and the replacement of MenC with meningococcal (Men) ACWY vaccine in adolescents, respectively [16].

This study aims to investigate the epidemiology of meningococcal disease in the UK during the period between 2007 and 2017 through the analysis of data collected from the UK Clinical Practice Research Datalink (CPRD) linked with data from the Hospital Episode Statistics (HES), and Office for National Statistics (ONS) mortality registry, including a description of the sequelae following meningitis disease for a more comprehensive assessment.

## Methods

### Study design

**Fig. 1:**
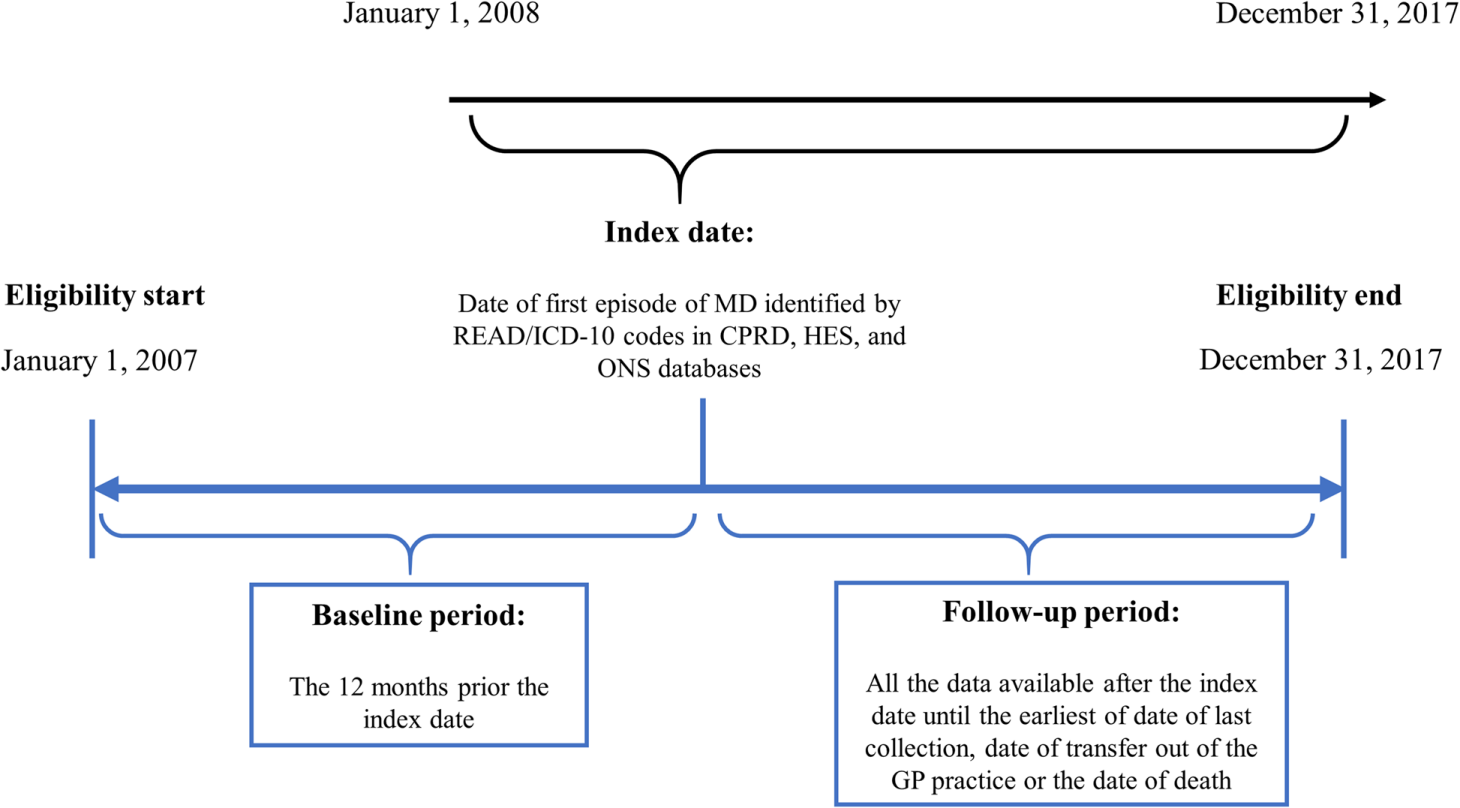
Study design. CPRD, UK Clinical Practice Research Datalink; GP, general practitioner; HES, Hospital Episode Statistics; ICD, International Classification of Diseases; MD, meningococcal disease; ONS, Office for National Statistics

This was a 10-year retrospective observational cohort study designed to describe the incidence and the Case-Fatality Rate (CFR) due to meningococcal disease, as well as the occurrence of sequelae in the UK between 2007 and 2017 using data from Clinical Practice Research Datalink (CPRD) GOLD, linked with data from the Hospital Episode Statistics (HES), and Office for National Statistics (ONS) mortality registry data. The CPRD is an ongoing primary care database of anonymised medical records from general practitioners, in the UK. Patients with an event of meningococcal disease were identified between 2008 and 2017 and were individually matched with up to four randomly selected controls based on age, gender, region, date of meningococcal disease diagnosis and follow-up duration. Index date was defined as the first meningococcal disease episode that occurred between 2008 and 2017. Controls were used for only the second part of the study, i.e., for the comparison of the occurrence of sequelae between cases and controls. A baseline period of 12-month of available data pre-index date and was required as an inclusion criterion for all patients aged ≥1 year. Follow-up period was defined by all reliable data available after index date until the earliest of the following events: date of last collection, date of transfer out of the general practitioner (GP) practice, or the date of death (Fig. 1).

### Study population/data source

Assessment of incidence and CFR of meningococcal disease included all patient records from January 1, 2008 to December 31, 2017 with a Read code (supplementary appendix) for meningococcal disease in CPRD or an International Classification of Diseases (ICD)-10 code (supplementary appendix) for meningococcal disease as the primary discharge diagnosis in the HES databases and an ICD-10 code for any mention of meningococcal disease as the causes of death in the ONS mortality database. The control group included patients identified in the CPRD, HES, and ONS databases without any records of meningococcal disease from January 1, 2008 to December 31, 2017. The meningococcal-related sequelae were assessed during the follow-up period using specific Read codes (supplementary appendix) and ICD-10 codes selected after review of the literature and categorized as per Table 1.

**Table 1:** Sequelae categories

Categories	Sub-categories	Types
Physical	Dermatological conditions	Skin scarring (including skin graft)
	Cardiovascular conditions	Symptoms consistent with Raynaud phenomenon, venous thrombosis, vasculitis, pericarditis, endocarditis, pericardiocentesis, and cardiac arrest
	Renal conditions	Renal failure (acute and chronic) and urinary failure
	Musculoskeletal deficiencies (bone, joint, muscle)	Arthritis, limb deficiency/deformities, amputation, arthralgia, and bone growth distortion
	Other physical conditions	Pulmonary condition, respiratory distress syndrome, sepsis, toxic shock syndrome, disseminated intravascular coagulation, coma, gangrene, diabetes insipidus, acute liver disease, sequelae of other specified infectious and parasitic diseases, and disorder of tooth development
Neurological	Sensory system deficits	Blindness and hearing loss (mild, moderate, severe, and profound)
	Motor deficits	Paralysis, cerebral palsies, muscle weakness, monoparesis, hemiparesis, movement coordination, spasticity, mobility problems, severe neuromotor-impairment, and balance impairment
	Communications disorders	Aphasia, general speech, and language and communication difficulties
	Intellectual disability	Mental retardation (IQ < 70), mild IQ loss (IQ 70–85), learning disabilities, and cognitive deficits
	Abnormal brain activity	Seizures (epileptic and non-epileptic), chronic headaches/migraine, dizziness and giddiness, and disorders of vestibular function
	Other severe neurological disorders	Hydrocephalus
Psychological/behavioural	Anxiety disorders	Generalized anxiety, separation anxiety, social anxiety disorder, and specific phobia
	Behavioural disorders	Conduct disorder
	Other psychological/emotional/behavioural disorders	Depression, post-traumatic stress disorder, disturbance of activity and attention, and other disorders of psychological development

Abbreviation: *IQ* intelligence quotient

### Study outcomes

Primary outcomes included overall incidence rate of meningococcal disease per 100,000 person-years assessed by year, age group, and diagnosis setting – hospital, emergency, outpatient, primary consultation and CFR (by year and age group). Age stratification included the age groups <1 year, 1 to 4 years, 5 to 14 years, 15 to 24 years, 25 to 49 years, 50 to 64 years, and ≥65 years. Additional outcomes included descriptive statistics for demographic characteristics, Charlson comorbidity index (CCI) score, high risk status (immunosuppression, active and passive smoking, and winter infections caused by respiratory syncytial virus, influenza, influenza like illness and pneumonia), deaths, and sequelae (supplementary appendix).

### Statistical analyses

Each study measure was summarized using unadjusted methods. Continuous measures were summarised by their medians and the interquartile range (IQR), along with their mean and respective standard deviation (SD). Categorical variables were summarized by numbers and proportions. The annual incidence and CFR due to meningococcal disease for each year of the study (2008–2017) were calculated per 100,000 person-years with the corresponding 95% confidence interval (95% CI) using the Poisson distribution. The rates for the entire 10-year study period were calculated as the average of the annual rates between 2008 and 2017. Demographic characteristics of patients with meningococcal disease were described at index date and at the end of follow-up period. For the assessment of disease sequelae, analyses were performed overall and by age group in both cases and matched controls. Descriptive statistics was provided for the analysis of sequelae (at least one sequelae) and by type of sequelae. Incidence risks, incidence rates, time between the index date of meningococcal disease and the occurrence of the first sequelae (time-to-event), were calculated. The incidence risks have been assessed as the number of patients with the sequelae of interest divided by the total number of patients at each time-point. The incidence rate was calculated as the number of first occurrences of each type of sequela during the follow-up period divided by the total aggregate person-time accrued by patients. Kaplan–Meier curves were depicted for the occurrence of sequelae. The *P*-value of log-rank test was computed to compare the survival distributions of cases and controls. Multivariate Cox models were used to adjust the hazard ratio (HR) of sequelae occurrence between cases and controls. Covariates included in the multivariate models were baseline demographic characteristics, CCI score, and the high-risk status. All analyses were performed using Pyspark and R.

## Results

### Incidence and mortality

The study included 640 IMD patients (median age: 7 years [range, 0–98 years]; male: 54.4%) with a diagnosis of meningococcal disease between 2008 and 2017. Overall, majority of the patients were diagnosed in a hospital setting (76.9%), but in those 25 to 49 years old, the diagnosis was made equally at the hospital and primary consultation settings. Over the study period, the median age at diagnosis increased, from 1 year in 2008 to 23 years in 2017. Analysis by age group showed a decrease in the occurrence of the disease in those aged <1 year (30.4–7.5%) and an increase in the occurrence of the disease in those >50 years (10.4–27.8%) (Table 2). During the study period, 45 patients died with a mention of meningococcal disease as cause. Mortality rate was slightly higher among females (55.6% vs 44.4%), toddlers (22.2%), and adults above 50 years of age (55.6%).

**Table 2:** Demographic characteristics of patients with meningococcal disease

Characteristics		2008		2009		2010		2011		2012		2013		2014		2015		2016		2017		2008–2017 (Average)	
		***N***	%	***N***	%	***N***	%	***N***	%	***N***	%	***N***	%	***N***	%	***N***	%	***N***	%	***N***	%	***N***	%
Total	All	125	100.0%	88	100.0%	75	100.0%	68	100.0%	67	100.0%	66	100.0%	43	100.0%	50	100.0%	40	100.0%	18	100.0%	64.0	100.0%
Gender	Female	60	48.0%	34	38.6%	41	54.7%	31	45.6%	32	47.8%	30	45.5%	21	48.8%	22	44.0%	15	37.5%	6	33.3%	29.2	45.6%
	Male	65	52.0%	54	61.4%	34	45.3%	37	54.4%	35	52.2%	36	54.5%	22	51.2%	28	56.0%	25	62.5%	12	66.7%	34.8	54.4%
Age (in years)	Median	1		7		3		4		6		6		3		17		21		23		7	
Age group	<1 year	38	30.4%	20	22.7%	20	26.7%	10	14.7%	13	19.4%	10	15.2%	8	18.6%	5	10.0%	3	7.5%	2	11.1%	12.9	20.2%
	1**–**4 years	46	36.8%	19	21.6%	22	29.3%	22	32.4%	17	25.4%	17	25.8%	11	25.6%	12	24.0%	9	22.5%	3	16.7%	17.8	27.8%
	5**–**14 years	12	9.6%	19	21.6%	9	12.0%	7	10.3%	11	16.4%	13	19.7%	2	4.7%	5	10.0%	3	7.5%	1	5.6%	8.2	12.8%
	15**–**24 years	6	4.8%	5	5.7%	7	9.3%	7	10.3%	7	10.4%	4	6.1%	5	11.6%	9	18.0%	6	15.0%	3	16.7%	5.9	9.2%
	25**–**49 years	10	8.0%	12	13.6%	9	12.0%	10	14.7%	8	11.9%	7	10.6%	5	11.6%	5	10.0%	8	20.0%	4	22.2%	7.8	12.2%
	50+ years	13	10.4%	13	14.8%	8	10.7%	12	17.6%	11	16.4%	15	22.7%	12	27.9%	14	28.0%	11	27.5%	5	27.8%	11.4	17.8%
Region	East Midlands	8	6.4%	2	2.3%	1	1.3%	1	1.5%	6	9.0%	1	1.5%	0	0.0%	0	0.0%	0	0.0%	0	0.0%	1.9	3.0%
	East of England	8	6.4%	7	8.0%	7	9.3%	7	10.3%	5	7.5%	2	3.0%	3	7.0%	4	8.0%	2	5.0%	3	16.7%	4.8	7.5%
	London	8	6.4%	17	19.3%	8	10.7%	12	17.6%	12	17.9%	15	22.7%	7	16.3%	6	12.0%	9	22.5%	3	16.7%	9.7	15.2%
	North East	6	4.8%	4	4.5%	0	0.0%	2	2.9%	3	4.5%	0	0.0%	0	0.0%	0	0.0%	3	7.5%	0	0.0%	1.8	2.8%
	North West	25	20.0%	19	21.6%	15	20.0%	16	23.5%	11	16.4%	12	18.2%	6	14.0%	12	24.0%	6	15.0%	5	27.8%	12.7	19.8%
	South Central	17	13.6%	7	8.0%	8	10.7%	3	4.4%	2	3.0%	5	7.6%	11	25.6%	9	18.0%	5	12.5%	1	5.6%	6.8	10.6%
	South East Coast	18	14.4%	10	11.4%	6	8.0%	9	13.2%	5	7.5%	5	7.6%	5	11.6%	7	14.0%	7	17.5%	4	22.2%	7.6	11.9%
	South West	18	14.4%	8	9.1%	12	16.0%	9	13.2%	12	17.9%	8	12.1%	5	11.6%	5	10.0%	3	7.5%	0	0.0%	8	12.5%
	West Midlands	14	11.2%	11	12.5%	13	17.3%	6	8.8%	5	7.5%	15	22.7%	5	11.6%	5	10.0%	4	10.0%	2	11.1%	8	12.5%
	Yorkshire & The Humber	3	2.4%	3	3.4%	5	6.7%	3	4.4%	6	9.0%	3	4.5%	1	2.3%	2	4.0%	1	2.5%	0	0.0%	2.7	4.2%
Setting of the index event	Primary healthcare	31	24.8%	21	23.9%	17	22.7%	18	26.5%	11	16.4%	15	22.7%	7	16.3%	13	26.0%	11	27.5%	3	16.7%	14.7	23.0%
	Hospital care	93	74.4%	67	76.1%	58	77.3%	50	73.5%	56	83.6%	51	77.3%	36	83.7%	37	74.0%	29	72.5%	15	83.3%	49.2	76.9%
	Null^a^	1	0.8%	0	0.0%	0	0.0%	0	0.0%	0	0.0%	0	0.0%	0	0.0%	0	0.0%	0	0.0%	0	0.0%	0.1	0.2%

^a^One patient diagnosed with only meningococcal disease was recorded in mortality data

**Fig. 2:**
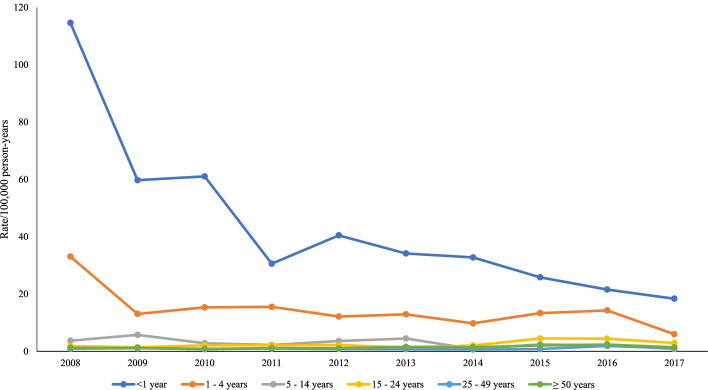
Annual incidence rates of meningococcal disease from 2008 to 2017 by age group

The incidence of meningococcal disease was higher in the beginning of study period compared with the end, with a decreasing trend over the years (Fig. 2). Annual incidence rates were the highest among those less than 4 years of age, but the incidence rate in these age groups decreased over the study period (from 114.62/100,000 person-years in 2008 to 18.37/100,000 person-years in 2017 [-83.97%] in those <1 year old and from 33.07/100,000 person-years in 2008 to 5.97/100,000 person-years in 2017 [-81.95%] in those 1–4 years old). In adolescents (15–24 years), the disease incidence increased over the study period (from 1.76/100,000 person-years in 2008 to 2.91/100,000 person-years in 2017 [+65.34%]) with a peak in 2015 and 2016 (4.48 and 4.39/100,000 person-years, respectively).

**Fig. 3:**
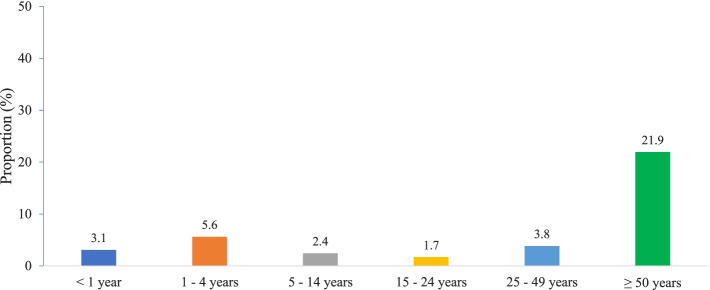
Case fatality rates from 2008 to 2017 by age group

There were no significant changes in CFR over the study years (CFR = 6.4% [95% CI, 3.6–11] in 2008 and 5.6% [95% CI, 1.2–21.5] in 2017). The highest CFR was reported in those 50 years and above of age. CFR was lower across the age groups (<1 year, 5–14 years, 15–24 years, and 25–49 years) compared with that in patients ≥50 years (Fig. 3).

### Occurrence of sequelae (case–control study)

**Fig. 4:**
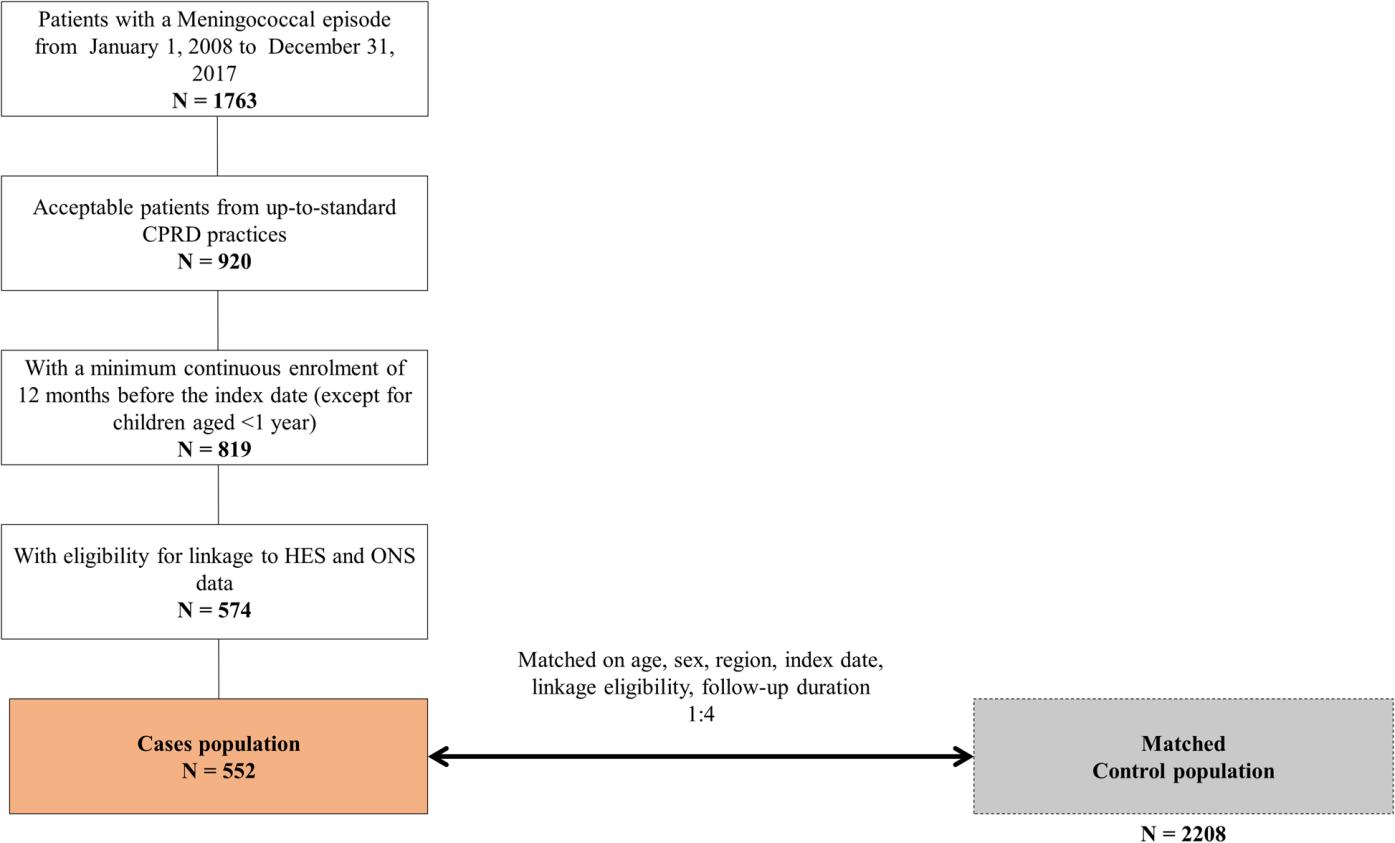
Patient selection (case–control study)

In total, 552 cases and 2208 controls with a mean follow-up time of 3.3 ± 2.7 years were included in the matched case–control part of this study (Fig. 4). Demographic characteristics of the matched population are displayed in Table 3. The severity of comorbid diseases was recorded and scored according to the CCI. Cases had a significantly higher frequency of a history of myocardial infarction (*P* = 0.026), congestive heart failure (*P* = 0.014), cerebrovascular disease (*P* = 0.037), pulmonary disease (*P* = 0.001), renal disease (*P* = 0.002), and cancer (*P* = 0.01) (Table 4). When considering CCI category at baseline, cases and controls had similar levels of comorbidities severity for all age groups, except in those >50 years old, where cases had significantly more severe comorbidities than controls. When considering all age categories together, cases had significantly more severe comorbidities than controls, but this was mostly driven by those >50 years old (data not shown).

**Table 3:** Demographic characteristics of patients in the case–control study

Characteristics		Case		Control	
		***N***	%	***N***	%
Total	Total	552	100.0%	2208	100.0%
Gender	Female	253	45.8%	1012	45.8%
	Male	299	54.2%	1196	54.2%
Age at index date (in years)	Average	19.95		19.95	
	Standard deviation	26.16		26.15	
Age group	<1 year	127	23.0%	508	23.0%
	1-4 years	141	25.5%	564	25.5%
	5-14 years	74	13.4%	296	13.4%
	15-24 years	50	9.1%	200	9.1%
	25-49 years	62	11.2%	248	11.2%
	50+ years	98	17.8%	392	17.8%
Region	East Midlands	16	2.9%	64	2.9%
	East of England	47	8.5%	188	8.5%
	London	86	15.6%	344	15.6%
	North East	12	2.2%	48	2.2%
	North West	115	20.8%	460	20.8%
	South Central	55	10.0%	220	10.0%
	South East Coast	63	11.4%	252	11.4%
	South West	67	12.1%	268	12.1%
	West Midlands	70	12.7%	280	12.7%
	Yorkshire and the Humber	21	3.8%	84	3.8%
Race	Missing	14	2.5%	294	13.3%
	Black African	3	0.5%	27	1.2%
	Black Caribbean	3	0.5%	12	0.5%
	Black other	3	0.5%	8	0.4%
	Indian, Pakistani, and Bangladeshi	9	1.6%	69	3.1%
	Other and mixed	23	4.2%	76	3.4%
	Other Asian	6	1.1%	38	1.7%
	White	491	88.9%	1684	76.3%

Abbreviation: *N* number

**Table 4:** Charlson comorbidities at baseline (case–control study)

**Charlson comorbidities**		**Cases**		**Controls**		***P*-value**^a^
		***N***	**%**	***N***	**%**	
*N*		552		2208		
Myocardial infarction		5	0.9%	5	0.2%	0.026
Congestive heart failure		7	1.3%	8	0.4%	0.014
Peripheral vascular disease		4	0.7%	9	0.4%	0.338
Cerebrovascular disease		7	1.3%	10	0.5%	0.037
Dementia		2	0.4%	12	0.5%	0.582
Pulmonary disease		36	6.5%	78	3.5%	0.001
Connective tissue disorder		3	0.5%	6	0.3%	0.327
Peptic ulcer disease		0	0.0%	0	0.0%	NA
Mild liver disease		3	0.5%	2	0.1%	0.05
Diabetes without complications		20	3.6%	26	1.2%	< 0.001
Diabetes with complications		0	0.0%	3	0.1%	0.992
Paraplegia		2	0.4%	2	0.1%	0.166
Renal disease		11	2.0%	13	0.6%	0.002
Cancer		12	2.2%	20	0.9%	0.01
Moderate or severe liver disease		2	0.4%	0	0.0%	0.992
Metastatic cancer		1	0.2%	7	0.3%	0.589
AIDS/HIV		0	0.0%	0	0.0%	NA
**Charlson comorbidity index**		**Cases**		**Controls**		***P*-value**
		***N***	**%**	***N***	**%**	
*N*	Total	552		2208		< 0.001^b^
CCI score	Average	0.27		0.12		
	Standard deviation	0.88		0.63		
CCI category	0	481	87.1%	2057	93.2%	< 0.001^c^
	1–2	50	9.1%	126	5.7%	
	3–4	17	3.1%	15	0.7%	
	≥5	4	0.7%	10	0.5%	

Abbreviations: *AIDS* acquired immunodeficiency syndrome; *CCI* Charlson comorbidity index; *HIV* human immunodeficiency virus; *N* number; *NA* not available

^a^Univariate conditional logistic regression

^b^Wilcoxon's test

^c^Univariate conditional logistic regression

**Fig. 5:**
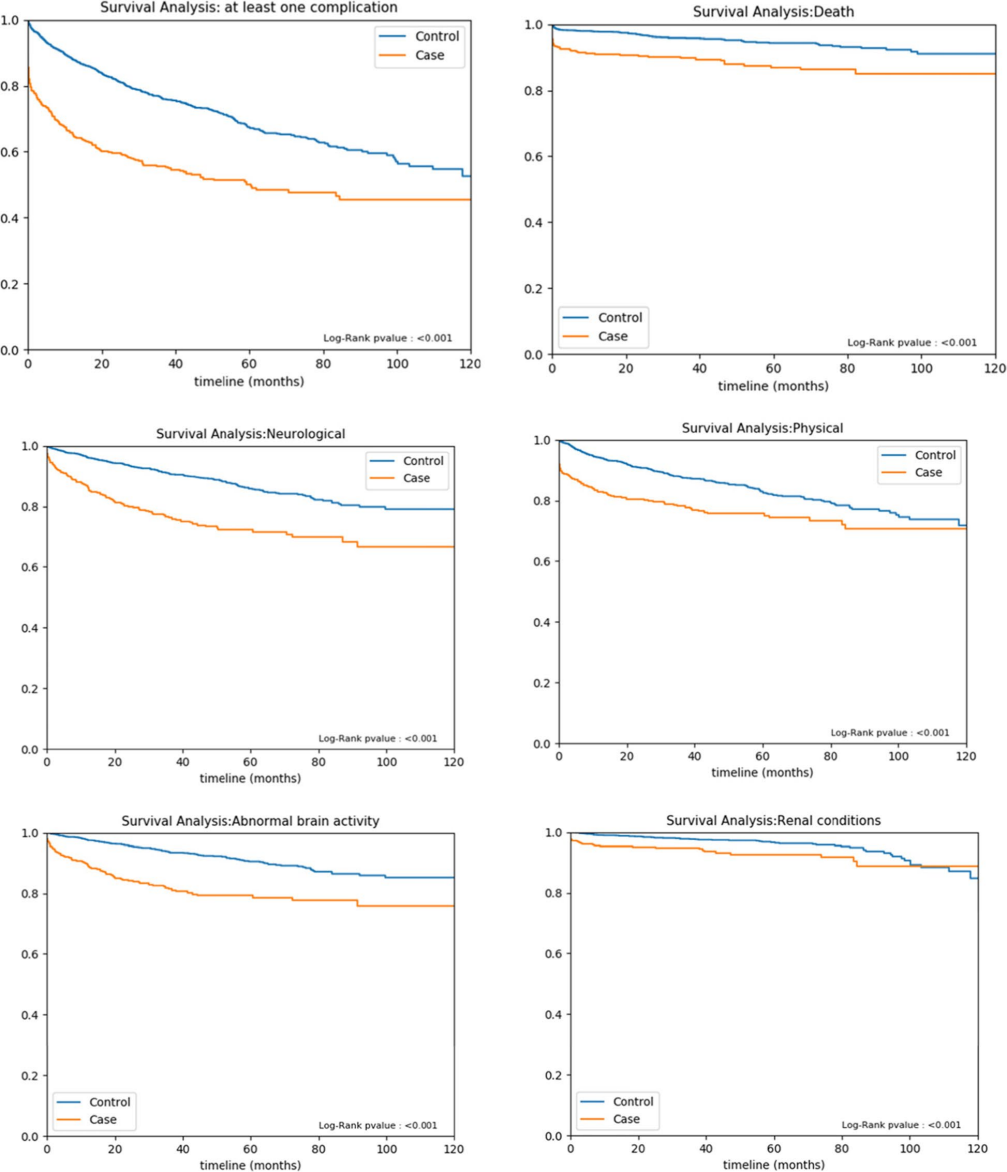
Kaplan–Meier curves of the occurrence of sequelae during the follow-up period

**Fig. 6:**
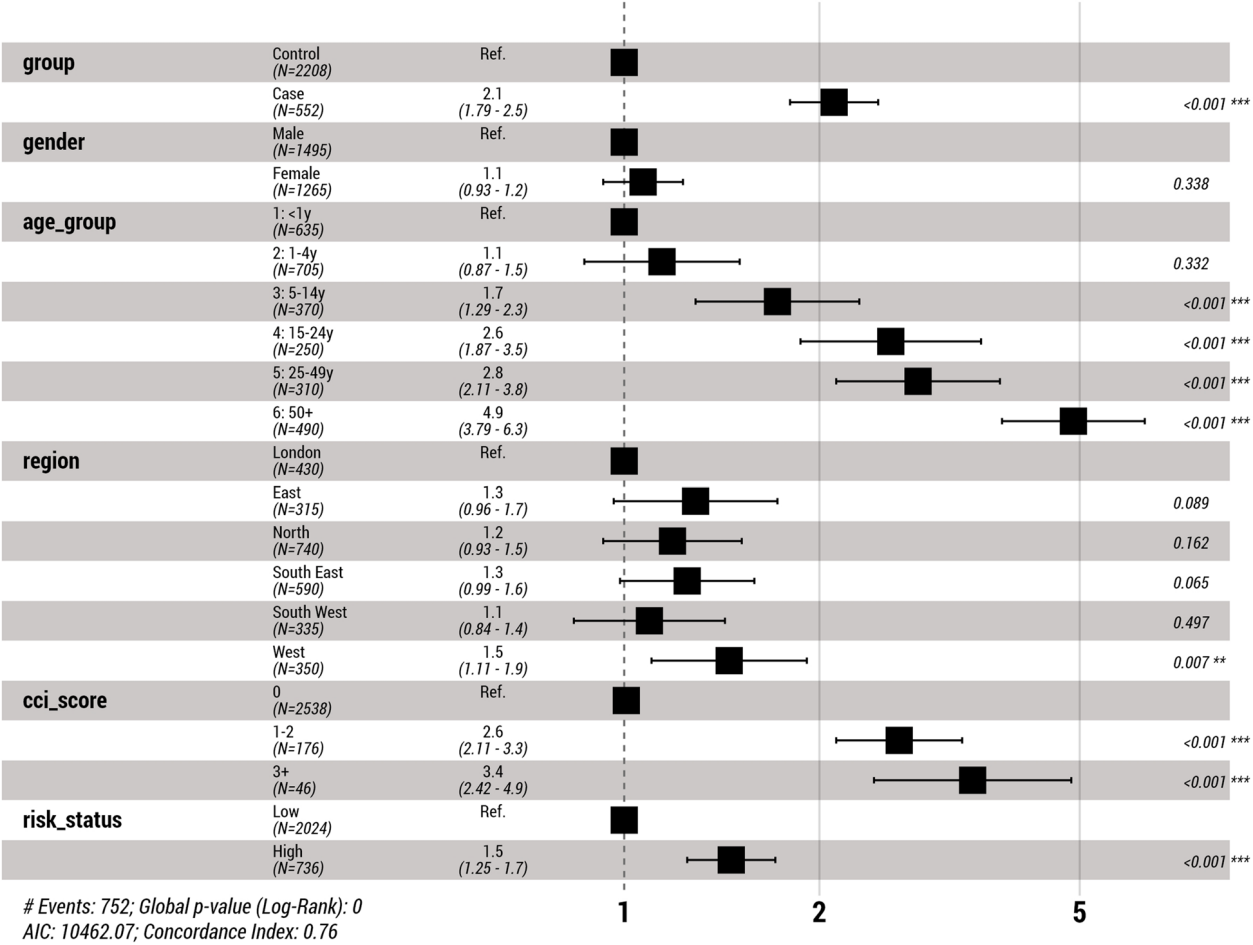
Hazard ratios for sequelae during the follow-up period using the multivariate cox regression model

During the follow-up period, for all age groups, cases had a higher probability of experiencing at least one sequela than controls (HR, 2.1; *P* < 0.001) (Table 5). In total, 61 (11.1%) cases died during the follow-up period. The overall probability of dying was significantly higher in cases than controls, mainly for those above 25 years of age. Except for infants, the probability of having a neurological sequela was consistently higher among cases than controls (HR, 2.39; *P* < 0.001). A higher probability of having a physical sequela was observed in cases than controls (HR, 1.63). Higher probability of developing renal conditions in infants and toddlers and cardiovascular conditions in young adults, was observed among cases compared with controls. A higher risk of psychological/behavioural sequelae was observed among cases than controls, but the difference was not statistically significant (HR, 1.46; *P* = 0.116) (Fig. 5). Psychological sequelae category took the longest time to develop with a median of 15.5 months in cases, and as high as 36.2 months in those <1 year old; it was followed by neurological sequelae (median, 8.5 months in cases) and physical sequelae (median, 1 month in cases). The risk increased with CCI score and was more than three times higher in those with the highest baseline scores (Fig 6).

**Table 5:** Sequelae observed during follow-up

Sequelae	Case (***N*** = 552)			Control (***N*** = 2208)			***P***-value
	***N***	Risk (%)	Rate (/1000 PY)	***N***	Risk (%)	Rate (/1000 PY)	
**At least one complication**	242	43.8 (39.8–48.0)	191.4 (168.1 - 217.1)	510	23.1 (21.4–24.9)	82.7 (75.7 - 90.2)	**< 0.001**
**Death**	61	11.1 (8.7–13.9)	33.3 (25.5 - 42.8)	93	4.2 (3.5–5.1)	12.6 (10.2 - 15.5)	**< 0.001**
**Neurological sequelae**	116	21.0 (17.8–24.6)	77.2 (63.8 - 92.6)	208	9.4 (8.3–10.7)	30.8 (26.7 - 35.3)	**< 0.001**
Abnormal brain activity	88	15.9 (13.1–19.2)	55.8 (44.8 - 68.8)	140	6.3 (5.4–7.4)	20.2 (17.0 - 23.9)	**< 0.001**
Communication disorder	4	0.7 (0.3–1.8)	2.2 (0.6 - 5.7)	9	0.4 (0.2–0.8)	1.2 (0.6 - 2.4)	0.329
Intellectual disability	19	3.4 (2.2–5.3)	10.6 (6.4 - 16.6)	46	2.1 (1.6–2.8)	6.4 (4.7 - 8.5)	**0.037**
Motor deficits	0	0.0 (0.0–0.7)	0.0 (0.0 - 2.0)	1	0.0 (0.0–0.3)	0.1 (0.0 - 0.8)	0.993
Sensory system deficits	14	2.5 (1.5–4.2)	7.9 (4.3 - 13.2)	28	1.3 (0.9–1.8)	3.9 (2.6 - 5.6)	**0.017**
Other neurological complications	3	0.5 (0.2–1.6)	1.7 (0.3 - 4.9)	2	0.1 (0.0–0.3)	0.3 (0.0 - 1.0)	0.050
**Physical sequelae**	115	20.8 (17.7–24.4)	76.8 (63.4 - 92.2)	268	12.1 (10.8–13.6)	40.7 (36.0 - 45.9)	**< 0.001**
Cardio/vascular conditions	10	1.8 (1.0–3.3)	5.6 (2.7 - 10.2)	16	0.7 (0.4–1.2)	2.2 (1.3 - 3.6)	**0.021**
Dermatological conditions	9	1.6 (0.9–3.1)	5.0 (2.3 - 9.5)	33	1.5 (1.1–2.1)	4.6 (3.2 - 6.5)	0.571
Musculoskeletal deficiencies	22	4.0 (2.6–6.0)	12.6 (7.9 - 19.0)	64	2.9 (2.3–3.7)	9.0 (6.9 - 11.5)	0.165
Renal conditions	34	6.2 (4.4–8.5)	19.3 (13.4 - 27.0)	64	2.9 (2.3–3.7)	8.9 (6.8 - 11.3)	**< 0.001**
Other physical conditions	78	14.1 (11.5–17.3)	49.4 (39.0 - 61.6)	135	6.1 (5.2–7.2)	19.5 (16.4 - 23.1)	**< 0.001**
**Psychological/behavioural sequelae**	26	4.7 (3.2–6.8)	15.0 (9.8 - 21.9)	66	3.0 (2.4–3.8)	9.3 (7.2 - 11.8)	0.039
Anxiety disorders	8	1.4 (0.7–2.8)	4.5 (1.9 - 8.8)	22	1.0 (0.7–1.5)	3.0 (1.9 - 4.6)	0.335
Behavioural disorders	0	0.0 (0.0–0.7)	0.0 (0.0 - 2.0)	0	0.0 (0.0–0.2)	0.0 (0.0 - 0.5)	NA
Other psychological/emotional/behavioural disorders	22	4.0 (2.6–6.0)	12.5 (7.9 - 19.0)	54	2.4 (1.9–3.2)	7.5 (5.7 - 9.8)	**0.044**

Abbreviations: *N* number; *PY* person-years

## Discussion

This study describes the epidemiology of meningococcal disease and the sequelae associated with meningococcal disease in the UK population using the CPRD database linked to HES and ONS data. The CPRD data have been extensively used for observational research, as it represents 7% of the UK population, and patients are broadly representative of the general population in terms of age, sex, and ethnicity [17].

The 10-year average annual incidence of meningococcal disease across all age groups in the study was approximately 2.7/100,000 population and decreased over the study period, which is consistent with the data published by Public Health England (PHE) in 2019 [18]. The incidence of the disease was higher in the beginning of our study, in infants and toddlers, and after 2012, no deaths were observed in these age groups. Although discrimination between serogroups was not possible, our data seemed to capture the impact of vaccination against MenC (introduced in 1999) and MenB (introduced in 2015) in the overall number of cases and deaths in the UK [19]. In adolescents (range: 15–24 years), the disease incidence increased over the study period (1.76 per 100,000 in 2008 to 2.91 per 100,000 in 2017) with a peak in 2015 and 2016 (4.48 and 4.39/100,000 person-years, respectively). The decline in the incidence for this age group after 2016 could be explained by the introduction of the quadrivalent ACWY conjugate vaccines into the routine immunization schedule for adolescents in the UK, which took place in 2015 [20].

In our study, the risk of developing at least one sequela was almost double among cases than controls (43.8% vs 23.1%, [HR, 2.1; *P* < 0.001]), which is aligned with published literature [2, 21], and was mainly age-dependent. The most frequent sequelae registered were neurological (21%), physical (21%), and/or psychological (5%). As follow-up durations may differ between studies comparisons should be made with caution.

The study presents some limitations inherent to the nature of the data extracted from the linked CPRD/HES/ONS databases, namely that data were not collected to address our particular research questions, and important variables, such as the vaccination status of the cases or serogroup distribution, were not available for research. The quality of the data on the long-term sequelae depends upon the retention rate of the patients in the CPRD/HES database, duration of follow-up for each patient, and healthcare resources being tracked in those databases. Some patients wouldn’t have had enough time to experience a long-term event which could have led to underestimation of some sequelae. We cannot exclude that some patients seek care outside of GP practices or hospitals captured in the assessed databases, which could have underestimated the number of cases with sequelae. Moreover, in our study cases had more comorbidities at baseline than controls, hence it is possible that the sequelae were caused due to comorbidities (for example, heart diseases) instead of IMD. Also, pre-existing conditions for the development of sequelae were not assessed, which might have underestimated some complications linked to exacerbation of some pre-existing conditions. In addition, as recognised by the World Health Organization (WHO) “defeating meningitis by 2030 roadmap” [22], there are limited data on the long-term impact of meningitis, and there is limited guidance on how to develop and conduct studies and surveys of sequelae, including its definitions.

## Conclusions

Our findings show that meningococcal disease still poses a significant burden in the UK with patients at an increased risk of developing sequelae which may be associated with additional social and economic burden. The data shows that the analysis of existing data (secondary use of data) could be a useful resource to complement data from the notification systems to better assess the true burden of the disease. Strengthening the prevention through optimisation of vaccination programs may assist in reducing the disease burden. Continuous monitoring of the disease remains an important tool in the prevention and control of this disease and will help in the evaluation of the immunization programs. It is of utmost importance that the monitoring of sequelae is an integral part of the surveillance of meningococcal disease. It should be noted nevertheless that the longitudinal follow-up of patients and the availability of data from different datasets can pose challenges. There is a need for better access to large healthcare databases and development of linkage methods at national level to help characterize the long-term sequelae that meningococcal disease can cause.

## Supplementary Information

**Additional file 1.**

## Data Availability

The data used for this study were obtained from the Clinical Practice Research Datalink (CPRD). The datasets generated during and/or analysed during the current study are not publicly available but are available from the corresponding author on a reasonable request.

## Abbreviations

CCI: Charlson comorbidities index
CFR: Case fatality rate
CI: Confidence interval
CPRD: UK Clinical Practice Research Datalink
DALY: Disability-adjusted life years
HES: Hospital Episode Statistics
HR: Hazard ratio
ICD: International Classification of Diseases
IMD: Invasive meningococcal disease
IQR: Interquartile range
Men: Meningococcal
ONS: Office for National Statistics
PHE: Public Health England
SD: Standard deviation
UK: United Kingdom
WHO: World Health Organization
YLD: Years of life lived with disability
